# The Structural Determinants behind the Epigenetic Role of Histone Variants

**DOI:** 10.3390/genes6030685

**Published:** 2015-07-23

**Authors:** Manjinder S. Cheema, Juan Ausió

**Affiliations:** Department of Biochemistry and Microbiology, University of Victoria, Victoria, BC V8W-3P6, Canada; E-Mail: mscheema@uvic.ca

**Keywords:** chromatin, epigenetics, histones, histone variants, nucleosome

## Abstract

Histone variants are an important part of the histone contribution to chromatin epigenetics. In this review, we describe how the known structural differences of these variants from their canonical histone counterparts impart a chromatin signature ultimately responsible for their epigenetic contribution. In terms of the core histones, H2A histone variants are major players while H3 variant CenH3, with a controversial role in the nucleosome conformation, remains the genuine epigenetic histone variant. Linker histone variants (histone H1 family) haven’t often been studied for their role in epigenetics. However, the micro-heterogeneity of the somatic canonical forms of linker histones appears to play an important role in maintaining the cell-differentiated states, while the cell cycle independent linker histone variants are involved in development. A picture starts to emerge in which histone H2A variants, in addition to their individual specific contributions to the nucleosome structure and dynamics, globally impair the accessibility of linker histones to defined chromatin locations and may have important consequences for determining different states of chromatin metabolism.

## 1. Histones, Canonical Histones, Histone Variants and Epigenetics

The name “*histon*” was coined in 1884 [1] to allude to the peptone nature of the chemical constituents that could, as a result of their rich composition in basic amino acids, be extracted from nuclei of tissues (Greek: *istos*) with dilute acids—a method still in use for histone isolation [2,3].

Histones represent the major chromosomal protein component of chromatin. Core histones and linker histones are the two main types of histones, categorized based on their structure and fundamental functions. Core histones (H2A, H2B, H3 and H4) are 100–140 amino acid long proteins that structurally consist of a histone fold domain (HFD) [4] flanked by intrinsically disordered N- and C- terminal regions. In H3 and H4, the C-terminal domains are very short (3–8 amino acids). These histones form the “core” around which approximately 200 bp of DNA are wrapped to form the fundamental repeating unit of chromatin called nucleosome [5]. Nucleosomes are linked to each other in the chromatin fiber through variable linker [6] DNA regions (approx. 10–100 bp ). Linker histones (H1 family) are 200–400 amino acid long proteins that bind to these regions and play an important role in the modulation of the chromatin fiber folding [7].

During replication of DNA in the S-phase of cell cycle, and in order to maintain the proper chromatin organization, there is a high demand for histone synthesis and deposition onto the newly synthesized DNA. This requires a quick transcription and translation of histone genes and, in metazoan animals, results in the encoding of RNAs that are not poly-adenylated and do not contain introns–presumably to reduce the post-transcriptional processing. The histones encoded by these genes are known as canonical histones [8].

**Table 1:** Vertebrate Histone Variant Gene Characteristics.

Histone	Number of introns	polyA+/−	Chromosome location
H2A.Z.1	4 introns	polyA+	4q23
H2A.Z.2	4 introns	polyA+	7p13
H2A.Z.2.2	4 Introns (Alternative Splicing)	polyA+	7p13
H2A.X	0 introns	polyA−/polyA+	11q23.3
H2A.B (H2A.Bbd)	0 introns in human	polyA+	Xq28
MacroH2A.1.1	10 introns (Alternative splicing)	polyA+	5q31.1
MacroH2A.1.2	10 introns (Alternative splicing)		5q31.1
	(9 coding exons)		
MacroH2A.2	8 introns (8 coding exons)	polyA+	10q22.1
TH2B	0 introns	polyA−	6p22.2
H2BFWT	2 introns	polyA+	Xq22.2
H3.3	2 introns	polyA+	1p42.12/17q25.1
CenH3	4 introns	polyA+	2p23.3
Human H1.0	0 introns	polyA+	22q13.1
Human H1.4	0 introns	polyA−	6p21.3
Human H1x	0 introns	polyA+	3q21.3
Chicken H5	0 introns	PolyA+	1
*M. surmuletus* PL-I	Unknown	PolyA+	Unknown

**Figure 1:**
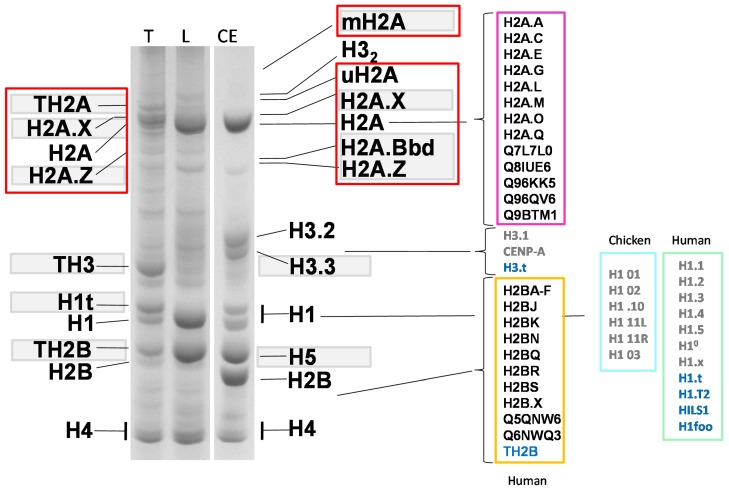
Acetic acid (5%) urea (5M) -triton (0.3%) polyacrylanmide gel electrophoresis of HCl-extracted histones from nuclei of: CE (chicken erythrocyte); L (rat liver) and T (rat testes). The replacement histone variants are highlighted with a grey background, and the different types for the canonical variants are also indicated. Members of the histone H2A family are highlighted by red squares to underscore the large number of variants within this family. Names in light blue on the right hand side of the image correspond to the germline variants. For clarity, the old histone nomenclature has been used in this figure. For equivalence to the newly unified phylogeny-based nomenclature, the reader is referred to [36].

In contrast to canonical histones, the term “histone variants” is globally used to describe those histones that are expressed throughout the cell cycle in smaller quantities; they often replace the canonical histones during chromatin metabolism, and hence are also referred to as “replacement variants”. See references [9,10] for a more detailed classification of these variants. Their genes often contain introns and, in comparison to canonical histones, the transcribed mRNAs are poly-adenylated (Table 1). It is possible to resolve most of the histone variants from their canonical counterparts by using acetic acid-urea-triton (AUT) polyacrylamide gel electrophoresis [11] (Figure 1).

In addition to histone variants, histone post-translational modifications (PTMs) and their “writers”, “erasers”, and “readers” [12] create a histone code [13] that has brought histones into the limelight of epigenetics [14]—an involvement they share with DNA methylation [15,16,17], and with the additional forms of this important DNA modification [18,19,20]. While a debate over the true epigenetic nature of the histone marks is still in progress [21,22], the molecular mechanisms involved in their maintenance during cell division [23,24,25,26,27] and the trans-generational transfer of the epigenetic marks [28,29] is starting to be elucidated [26,30]. Besides, there is growing evidence in support of histones’ epigenetic role and—more specifically—the role of histone variants [31,32,33,34,35].

## 2. Histone Variants as Epigenetic Markers

Several recent reviews have highlighted the epigenetic role of histone variants [33,34,37,38]. Furthermore, they have been shown to play an important role in the progression of cancer to different stages [39,40,41,42,43,44].

CenH3 (aka CENP-A) is a typical variant that exemplifies the epigenetic role of histone variants. One of the most dramatic compositional and conformational changes of chromatin in metazoan organisms takes place during the late stages of the spermatogenesis, after meiosis (spermiogenesis). During this time, in many invertebrate and vertebrate organisms (but not all), the nucleosomal chromatin organization disappears, and most of the histones are replaced by small arginine-rich chromosomal proteins known as protamines [45]. In mammals [46], and in some insects such as *Drosophila* [47], more than 95% of the histones are displaced by protamines. Interestingly, the centromeric regions are only partly affected by this drastic chromatin remodelling [48], and CenH3 (CID in *Drosophila*) is retained at these centromeric regions [49,50]. This ensures a trans-generational inheritance of the CenH3, which is key to the epigenetic process. Moreover, the formation or acquisition of new neo-centromeric nucleosomes appears to be more dependent on this variant than on the underlying DNA sequence [34].

An important part of the histone variant epigenetic mechanisms are mediated by chromatin remodeling complexes or transcriptional effectors that specifically interact with them. However, in this review we focus on the structural features of the histone variants and the ensuing chromatin alterations that directly underlie their epigenetic contribution. We will be following the recently proposed unified phylogeny-based nomenclature for histone variants [36].

## 3. The Structural Epigenetic Importance of the H2A-H2B Dimer

Structurally, dissociation of the histone H2A-H2B dimer results in the exposure of a region around the DNA pseudo-dyad axis of symmetry in the nucleosome [51]. This results in enhanced micrococcal nuclease digestion at this region, producing two 70 bp DNA fragments that lie at each side of the pseudo-dyad axis of symmetry. This important observation was proposed to explain the split-in-half nucleosome (sub-nucleosome) structures that were observed upon transcriptional activation of the heat-shock inducible *HSP82* gene in yeast [52]. Interestingly, a recent genome-wide analysis in budding yeast has provided further support to the idea of a widespread presence of these (and other) sub-nucleosomal particles [53]. Thus, the association of the histone H2A-H2B dimer with the nucleosome, including its many different variants [31], plays a critical role in nucleosome dynamics [54] and functional adaptability [55]. However, the structural role of the different H2A variants contributing to this dynamic has not always been straightforward, and, in several instances, has resulted in a new epigenetic paradigm.

### 3.1. H2A.Z Variants: The Structural and Functional Role of the Many Subtypes of H2A.Z

The structural role of H2A.Z in the regulation of transcription—and its function in general—remains highly controversial and puzzling. H2A.Z has been described as being present in both repressed and actively transcribing regions of chromatin [56,57]. The emerging view suggests that the functional role of this variant is chromatin context-specific, and can be considered as a transcriptional control rheostat [58].

**Figure 2:**
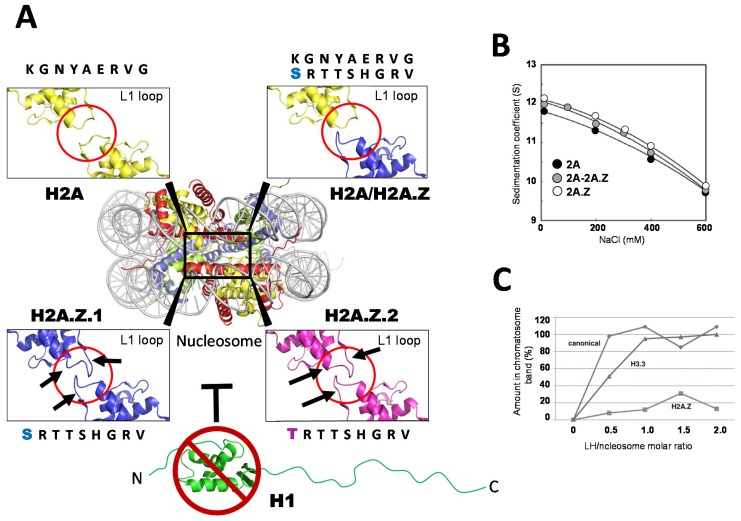
(**A**) Contacting regions, involving loop 1, of the histone fold for the two H2A histones present in the nucleosome. The details are shown for a homotypic nucleosome consisting of two canonical H2A histones (H2A), a heterotypic nucleosome consisting of a canonical H2A and an H2A.Z histone variant (H2A/H2A.Z), and two homotypic nucleosomes consisting of two H2A.Z.1 (H2A.Z.1) or two H2A.Z.2 (H2A.Z.2) variants. The loop 1 regions are highlighted in red and the black arrows to indicate the sites where differences are observed. The images were prepared from the crystallographic structures of the canonical histone nucleosome [78], the H2A.Z-containing nucleosome [79], and the H2A.Z.1 and H2A.Z.2 containing nucleosomes [67]. The inability of histone H1 to bind to the H2A.Z-containing nucleosome (see (**C**)) is indicated (┬); (**B**) Dependence of the sedimentation coefficient of reconstituted canonical nucleosomes (2A), homotypic nucleosomes consisting of H2A.Z (2A.Z), and heterotypic nucleosomes consisting of H2A and H2A.Z (2A-2A.Z), in Svedberg units (S) as a function of the ionic strength concentration (NaCl) in a 20 mM Tris-HCl (pH7.5) 0.1 mM EDTA buffer [64]; (**C**) Binding of histone H1 (linker histone, (LH)) to the nucleosome (chromatosome formation) as a function of the molar amount of linker histone (LH) per mol of nucleosome [68]. The titration was carried out using reconstituted nucleosomes consisting of either canonical histones, H3.3, or H2A.Z.

In general, nucleosomes containing a double copy of this variant (H2A.Z-homotypic nucleosomes) are slightly more compact and stable than their canonical counterpart [59,60]. However, several structural possibilities can be envisaged that might presumably account for the manifold functionality of this variant (Figure 2A). One possibility is its homotypic- *vs.*-heterotypic H2AZ-containing nucleosome’s (a nucleosome containing one H2A.Z and a canonical H2A histone) existence in the cell [61,62]. Indeed, homotypic H2A.Z nucleosomes are observed to be preferentially enriched downstream of active gene promoters and intron-exon junctions, whereas heterotypic H2A.Z nucleosomes are found elsewhere [63]. It has been proposed that the slight change in the organization of loop 1 (L1) between canonical H2A and H2A.Z (Figure 2A) could result in steric hindrance that would destabilize the nucleosome. However, heterotypic nucleosomes have been successfully reconstituted [64,65], and exhibited stability and hydrodynamic behavior very similar to that of their homotypic counterparts (Figure 2B) [64].

A second possibility is the existence of two different histone H2A.Z subtypes (H2A.Z.1 and H2A.Z.2). In the past, H2A.Z.1 was conventionally known as H2A.Z, whereas H2A.Z.2 was named H2A.V and thus, until very recently, H2A.Z was supposed to be a unique, single-copy histone variant. In vertebrates, the two subtypes differ only by three amino acids but have a completely different gene sequence [66]. Previously, one amino acid difference in H2A.Z (replacement of an S in H2A.Z.1 by a T in H2A.Z.2) (Figure 2A) has been shown to affect the conformation of the flexible L1 domains of the HFD (Figure 2A) [67] but has not displayed any effect on the salt-dependent nucleosome stability [67]. Despite the small changes in L1 loop of H2A.Z variants (Figure 2A), the mutated residues of the loops have been shown to be responsible for the significant difference in chromatin association-dissociation dynamics demonstrated by FRAP [67]. This is an important observation that may contribute to the unresolved issue of dual functionality.

Regardless of the subtype differences, H2A.Z has been shown to prevent the interaction of linker histones (histone H1) with the nucleosome (Figure 2 C) [68]. The increase in accessibility of the nucleosomes containing H2A.Z and H3.3 to restriction enzymes observed *in vivo* [69] may be a reflection of this and also of the frequent co-existence of these two variants within the same nucleosome [70]. The absence of linker histones in these nucleosomes when within the proximity of the transcription start site (TSS) may facilitate the accessibility of trans-acting factors, such as transcription factors and hormone receptors, to these regions.

While the direct structural features responsible for the different functions of H2A.Z are yet to be elucidated, they could be the result of the different PTMs associated with this variant. For instance, it has recently been shown that mono-ubiquitinated H2A.Z is associated with facultative heterochromatin, and is also associated with the human female X-chromosome inactivation [71], while acetylated H2A.Z is found at promoters of actively transcribing genes genome-wide [72,73]. As a matter of fact, expression of a non-acetylatable form of H2A.Z in myoblasts has been shown to block myoblast differentiation [74]. To add to this complexity, an alternatively spliced variant of H2A.Z.2 has recently been described (H2A.Z.2.2) [75,76]; it is 14 amino acids shorter at the C- terminal domain. This histone variant is only present in primates, where it is preferentially found in the brain. This truncated version of H2A.Z.2 has been shown to destabilize the histone octamer, as well as the nucleosome [75]. This instability is not surprising, as it had long been shown that removal of the last 15 N-terminal amino acids of canonical histone H2A by an endogenous protease reduced the association of H2A-H2B dimer with the histone H3-H4 tetramer [77].

One of the structural issues regarding H2A.Z that requires attention is: the drastic instability of the H2A.Z-H2B dimer, that has been repeatedly observed *in vitro* [60,80]. The functional relevance of such an intriguing observation still remains obscure, but it contrasts with the modestly positive effect of H2A.Z on the NCP stability as explained above.

### 3.2. H2A.X: A Variant Guardian of the Genome

Histone variant H2A.X is expressed evenly throughout the cell cycle [81] in a poly (A+) and poly (A−) manner [82], which places it in a very unique position between canonical and variant histones. In metazoans, this variant is present—on average—in approximately one in every ten nucleosomes [83].

From a phylogenetic perspective, H2A.X appears to have co-evolved with canonical histone H2A, having recurrently appeared several times throughout the course of evolution [83]. This variant has a unique C-terminal sequence (…SQ(F/D)(LYFV)-COOH) which is characteristically phosphorylated (aka γ-H2A.X) as a result of DNA damage hence the name “histone guardian of the genome” [84]. However, in comparison to canonical H2A, this variant creates a specific chromatin organization which, in addition to the DNA damage response, allows it to participate in many other cell type-specific functions [85]. The functional role of γ-H2A.X has long been known to generate a docking domain for the recruitment and interaction of DNA repair factors [83]. It also assists with the recruitment of cohesin in order to stabilize the chromosome surrounding the broken ends of the DNA [34,86].

In contrast to the well-understood functional properties of H2A.X, the structural implications of this histone—particularly in relation to its characteristic C-terminal phosphorylation end required for the conformation of chromatin—have been quite controversial. Using yeast mutant as a model, it was initially shown that phosphorylation of the SQEL C-terminal end resulted in a decreased chromatin compaction [87], yet more recently phosphorylation was deemed to have no effect on either chromatin folding or nucleosome stability [88]. Our lab, however, using H2A.X phosphorylation mimetics and H2A.X phosphorylated with DNA-PK in mammalian cell lines, has shown that nucleosomes reconstituted with this variant are unequivocally de-stabilized, an effect that it is enhanced by phosphorylation of the N-terminal end of H2A.X. Moreover, whilst H2A.X does not abrogate the binding of histone H1 to the nucleosome, it impairs its binding in a way that it is enhanced by its C-terminally phosphorylated form [89]. The de-stabilizing properties of this variant can be partly responsible for the well-documented instability of the yeast nucleosome [90], where H2A.X is the main canonical H2A component of its genomic chromatin.

### 3.3. H2A.B: A Sperm-Specific Histone Variant with Potential Implications for Transcription and Cell Proliferation (?)

Histone H2A.B, previously known as H2A.Bbd, was initially identified by Chadwick and Willard in 2001 through a bioinformatics search of human ESTs with homology to H2A. A cDNA, encoding 115 amino acid proteins (with 48% identical to canonical H2A), was obtained. Different human cell lines were transfected with C-terminal GFP-myc tagged versions of H2A.B containing plasmids. The stable transfected myc-tagged version was purified with the nucleosome fractions on a sucrose gradient, and the GFP-tagged version was excluded from the Barr Body; hence, it was initially called H2A.Bbd [91].

Interestingly, it was not until almost 10 years later that the native form of H2A.B was identified as a histone H2A variant that plays an important role during spermatogenesis [92,93] and is retained in mature human sperm [92,94]. The functional role of the H2A.B in spermiogenesis is not clear, but the variant appears in elongating spermatids at a time when histones start being replaced by protamines, and histone H4 is maximally acetylated [92]. This would suggest a potential involvement in the facilitation of the histone-to-protamine transition that takes place at this stage. However, the retention of H2A.B in mature sperm would rather suggest a function in demarcating genes that is important for embryo development after fertilization [95]. More recently, ectopically expressed H2A.B has shown that the protein is associated with active transcription, mRNA processing [96], and is transiently enriched at sites of DNA synthesis [97]. Moreover, H2A.B is expressed in some Hodgkin’s lymphoma cell lines, with cells expressing higher levels of H2A.B displaying shorter doubling time [97]. This suggests a potentially intriguing involvement of H2A.B in cell proliferation.

**Figure 3:**
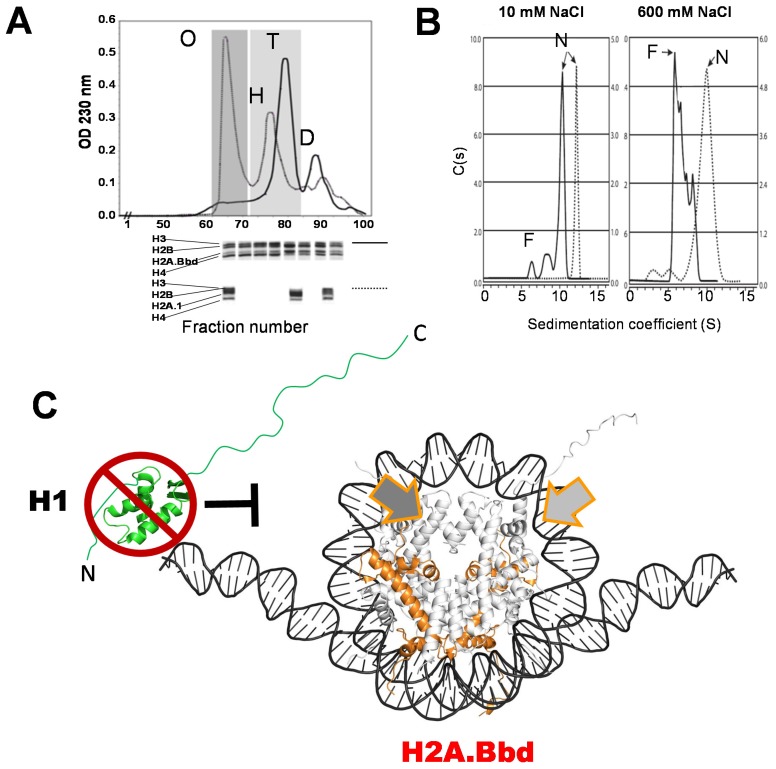
(**A**) Stability of the histone core octamer consisting of canonical H2A (solid line) or histone H2A.Bbd (dotted line) in the presence of 2M NaCl, as determined by gel filtration chromatography on a Sephacryl S-300 HR resin. O: octamer ((H3-H4)_2_.2(H2A-H2B)), H: hexamer ((H3-H4)_2_. (H2A-H2B)), T: tetramer ((H3-H4)_2_.), D: dimer ((H2A-H2B)/(H2A.Bbd-H2B)). The histones eluting with the different elution peaks are visualized in the SDS-PAGE shown underneath; (**B**) Ionic-strength-dependent stability of reconstituted NCPs containing H2A.Bbd (dotted line), in comparison to NCPs consisting of canonical H2A (solid line) as determined by sedimentation velocity analysis in the analytical ultracentrifuge. F: free (dissociated) DNA, N: nucleosomes; (**C**) Model for the H2A.Bbd (orange) structure of the H2A.Bbd-containing NCP, based on the crystallographic image of the nucleosome [78,98] and on the hydrodynamic characteristics [99,101] of the particle. The wide arrows indicate the region corresponding to the C-terminal domain of canonical H2A, which is missing in H2A.Bbd. The inability of histone H1 to bind to the H2A.Bbd-containing nucleosome [102] is indicated.

In the meantime, a plethora of structural characterizations were performed that included the crystallization of an H2A.B-containing nucleosome [98]. It was initially shown that H2A.B affected the interaction of the H2A-H2B dimer with the H3-H4 tetrasome so that no histone octameric complexes could be formed in solution (Figure 3A) [98]. Although it is possible to prepare nucleosomes using an equimolar concentration of H2A.B, H2B, H3, and H4 in the presence of DNA [99], these nucleosomes exhibited a significant salt-dependent instability ((Figure 3B) [100]). This instability brings to mind the H2A.Z.2.2 octamers and nucleosomes (see section H2A.Z variants). Like H2A.Z.2.2, histone H2A.B lacks the last 19 C-terminal amino acids corresponding to the canonical form. By looking at different biophysical characterization studies [99,101], it is very clear that the H2A.B-containing NCP adopts an extended structure (Figure 3C) in which approximately 13–15 nucleotides at each flanking site of the NCP are very flexible and detachable. Moreover, like histone H2A.Z, the presence of H2A.B in nucleosomes abolishes the binding of histone H1 [102]. A highly dynamic open conformation would be in agreement with the functional implications of this histone variant described above.

### 3.4. MacroH2A: The Longest Most Variable Histone Variant, Indispensable for Survival

MacroH2A is the longest and most structurally diverse histone variant, a property it shares with the long linker histone-related PL-I sperm proteins of some invertebrates [45]. MacroH2A consists of an NTD with a 60% sequence similarity to canonical DNA followed by an approximately 60 amino acid linker region, connected to an approximately 200 amino acid C-terminal globular non histone domain (NHD). Despite its uniqueness amongst histones, the variant is dispensable for survival, but knockout mice exhibit impaired reproductive efficiency [103]. Like H2A.B, it is less abundant (approximately 1 every 30 nucleosomes [104]), and, like H2A.Z, it consists of several isoforms: macroH2A.1.1 and macroH2A.1.2, the products of different alternative splicing, and macroH2A.2, encoded by a different gene [105,106].

Although initially identified as a repressor of the inactive X chromosome in mammalian females [105], and found to be present in several heterochromatin regions of the genome [107], it appears that functionally this variant can play both a negative and a positive role in the regulation of transcription [108]. Hence, its role at the gene expression level should be considered that of a regulator. The variant has also been shown to be involved in many cancer types [43]. The macro domain of macroH2A.1 is able to bind to NAD+ metabolites such as poly (ADP-ribose) [109,110]; however, the true functional implications of this ability remain largely unknown.

**Figure 4:**
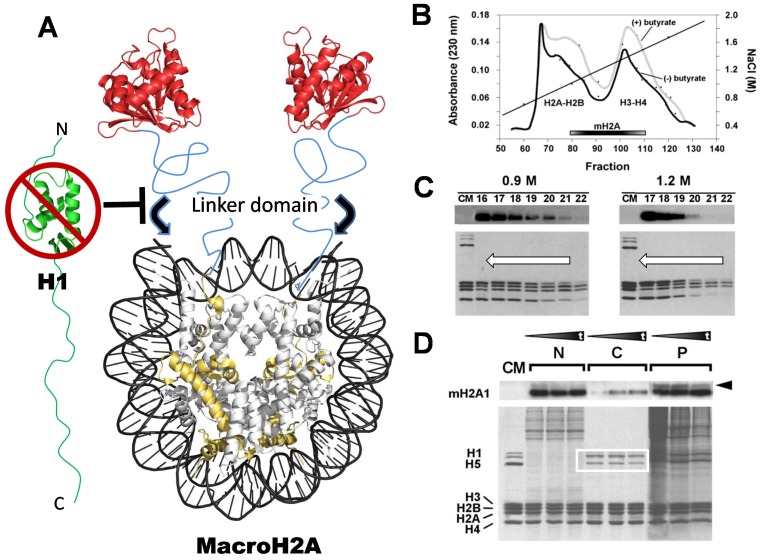
(**A**) Schematic representation of the macroH2A-containing nucleosome, based on the crystallographic structure of the N-terminal histone domain of macroH2A (gold) and on the crystallographic structure of the C-terminus of the macro domain (red) [111]. The tertiary structure of the linker domain region (depicted in blue) is not known, but it likely corresponds to an intrinsically disordered domain. The black arrows indicate the additional protection of DNA (approximately 10 bp at the entry and exit site of the DNA into the NCP by this linker region [112]). The inability of histone H1 to bind to the macroH2A-containing nucleosome (see (**D**)) is indicated; (**B**) Hydroxyapatite chromatography salt (NaCl) elution profiles of macroH2A, from chromatin extracts obtained from HeLa cells treated with or without sodium butyrate (to enhance global levels of histone acetylation). The elution of the H2A–H2B dimers, the H3-H4 tetramers, and also that of macroH2A, are indicated [113]; (**C**) MacroH2A western blot analysis of fractions (numbers) obtained from nucleosomes run on (5%–20%) sucrose gradients in the presence of 0.9 and 1.2 M NaCl. The SDS-PAGE analysis of the fractions is shown underneath. The arrows indicate the direction of the sedimentation. CM is chicken erythrocyte histone standard [110]; (**D**) MacroH2A western and SDS-PAGE analysis of chicken liver chromatin digested at increasing times, with micrococcal nuclease (black triangles). The digested chromatin thus obtained was fractionated according to a method initially described by Ada Olins *et al.* [114], which allows its separation into histone H1 depleted (N), histone H1-containing (white square) (C), and an insoluble (P) fractions [110].

The crystallographic structure of the nucleosome containing the macroH2A NTD, and of the macro CTD [111], have already been obtained; Figure 4A provides a model representation of the overall structure of the macroH2A-containing nucleosome. This image underscores the elongated shape of the macroH2A-containing nucleosome, which is clearly supported by the hydrodynamic characteristics as determined by sedimentation velocity [113]. In the context of native chromatin, macroH2A interacts more tightly than canonical H2A—as indicated by its later salt elution from chromatin that has been adsorbed onto hydroxyapatite (Figure 4B) [113]. The stronger binding to chromatin appears to be the result of a tighter interaction of the macroH2A-H2B dimer with the rest of the histone core, which confers a higher ionic strength-dependent stability to the nucleosome—as determined by sucrose gradient fractionation of nucleosomes in the presence of increasing salt concentrations, within the range at which the H2A-H2B dimer dissociates from the NCP (Figure 4C) [110].

Analysis of the chromatin distribution of macroH2A in relation to linker histones, using an approach based on the Olin’s method of micrococcal nuclease-digested chromatin fractionation (Figure 4 D) [110], provides evidence for a mutually exclusive relationship between histone H1 and this variant. Interestingly, it seems that the linker region of the macroH2A may substitute for the role of linker histones in enhancing chromatin folding in a way that is modulated by the macro domain [112,115].

## 4. Histone H2B Variants for the Germline

Two variants of histone H2B (TH2B and H2BFWT) are present in the mammalian germline, whereas H2BFWT [116] is present only in the male germline of primates, and TH2B together with TH2A are present in both the male and female germlines. TH2B is expressed quite extensively throughout spermatogenesis, starting in early spermatocytes and replacing most of canonical H2B in elongating spermatids, where it is believed to play a role in facilitating the replacement of somatic histones by protamines [117]. However, some of this variant is preserved in the mature sperm, at least in humans [118], which marks genes that are important for the events that take place immediately after fertilization [95]. The preservation of TH2B in mature sperm emphasizes the epigenetic role of this histone variant. H2BFWT binds to telomeric DNA sequences [116], but the native functional role of this histone variant is still unclear.

TH2B was the first testes-specific histone variant ever described [119], and represents the most abundant H2B in testis (Figure 1). It is 127 amino acids long and is highly homologous (89%) to H2B. By contrast, H2BFWT is 175 amino acids long and has only 70% amino acid similarity with H2B [116]. The largest extent of amino acid sequence diversity for both proteins takes place at their N-terminal end. Indeed, the H2BFWT N-terminal tail has a 42 amino acid extension, which is not present in canonical H2B [116].

The circular dichroism spectrum of TH2B reveals an increase in the α-helical content of the N-terminal region of TH2B when compared to canonical H2B. Similarly to H2A.B, the histone octamer containing TH2B is more unstable than the canonical histone octamer in 2 M NaCl solution, but it is able to reconstitute NCPs in the presence of DNA. The nucleosomes prepared *in vitro* in this way have an undistinguishable conformation to those containing canonical H2B, and the presence of this variant does not affect their ability to bind linker histones [120]. The structural properties of H2BFWT have been less extensively studied, but the presence of this variant does not appear to affect the association-dissociation efficiency of the H2BFWT-H2A dimer from the nucleosome, either *in vitro* or *in vivo* [121].

## 5. Histone H3 Variants: H3.3 and CenH3

Of the several histone H3 variants [33,122], we focus on H3.3 and CenH3, the structure and function of which have been more extensively studied in recent years [34].

### 5.1. H3.3: A Transcriptional Histone Mark that Accumulates with Age

In recent years, a lot of attention has been paid to histone H3.3 variant, due to its involvement in the regulation of transcription. There are many function-related studies on this variant [33,34,123,124,125]; however, we would like to briefly touch on an aspect that has been often overlooked. Despite its connection to gene activity, this histone variant quite counter-intuitively, and like histone H1.0 (see below), accumulates with age—and the rate of accumulation varies between tissues. This is a phenomenon that was described a long time ago [9], and has only been very recently revisited [126]. Thus, while in adult mouse thymus, spleen, and intestinal mucosa, H3.2 is the prevalent variant, in kidney and liver, H3.3 is the most abundant. The amount of H3.3 and H1.0 in tissues increases with increasing age, and they are both translated from poly-adenylated mRNAs (Table 1). These changes are paralleled by changes in the canonical histone H2A and H2B subtypes 1 and 2. H2B.1, H2A.2 and H2A.X increase as H3.3 increases with mouse age, and levels of H2B.2 and H2A.1 decrease [9]. Similar changes have been observed in rat cortical neurons [127], suggesting that in slowly or non-dividing cells, poly-adenylated histone variants are used to replace damaged canonical histones as a result of the wear and tear of the nucleus’s metabolic activities. However, the significance and implications of all this remain obscure.

**Figure 5:**
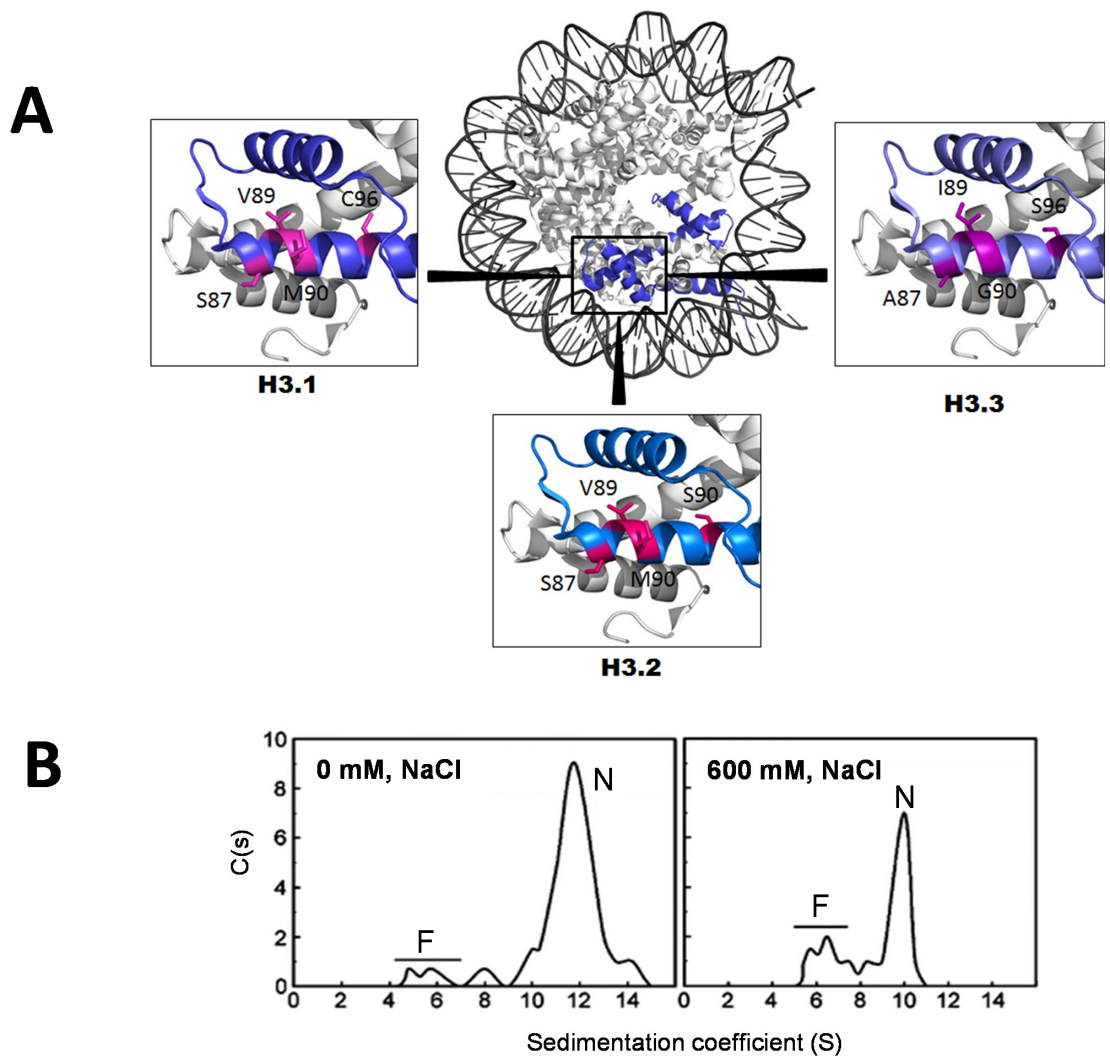
(**A**) Figures were obtained from the crystallographic structures of the H3.1 [134], H3.2, and H3.3-containing nucleosomes [128]. The amino acid residues where the sequences differ in the α2 helix of the histone fold are shown in fuchsia. The generic image of the nucleosome is from the early crystallographic analysis by Luger *et al.* (1997) [78]; (**B**) Ionic strength (NaCl)-dependent stability of reconstituted nucleosome core particles containing H2A.Z and H3.3 histones, as visualized in the analytical ultracentrifuge [68]. D: free DNA, N: nucleosomes, s_20,w_: sedimentation coefficient corrected for standard water and 20 °C conditions. The DNA used in the nucleosome reconstitutions was 155 ± 5 bp random sequence DNA fragments purified from chicken erythrocyte nucleosome core particles [135].

From a structural perspective, histone H3.3 differs in five amino acids from canonical H3.1, and in four from H3.2. Serine 31 at the NTD, and A87, I89, and G90 at α2 helix of the HFD of H3.3, replace A31, S87, V89, and M90 of both H3.1 and H3.2 respectively. Serine 96 replaces C96 at α2 helix of the HFD of H3.1. The structural differences imparted by these changes in the nucleosome are shown in (Figure 5B) [128]. These relatively small structural changes agree with the hydrodynamic properties of the H3.3-containing NCP, which exhibits a salt-dependent variation in the sedimentation coefficient and stability, which are indistinguishable from those of the canonical NCP [68]. Nevertheless, despite the few amino acid differences between H3.3 and H3.1, the former is used for replication-independent nucleosome incorporation, has specific chaperones (DAXX and HIRA) [129,130], and has a variant-specific “reader” of its K36me3 (BS69/ZMYND11), which is a regulator of intron retention during RNA splicing [131].

An important part of H3.3 in the cell is often found associated with H2A.Z-containing nucleosome [132]; therefore, it is important to analyze the effects this dual presence may exert on the nucleosome structure. Figure 5B shows the salt-dependent stability of nucleosomes reconstituted *in vitro* using purified, recombinantly expressed H3.3 and H2A.Z. These nucleosomes exhibit an identical stability as observed in reconstituted nucleosomes consisting of H3.1 and H2A.Z, or of canonical histones [68]. These results are important in view of the recent observations made *in vivo*, which suggested that nucleosomes containing both variants exhibit an unusual degree of instability in the cell setting [133]. Yet, the results shown in Figure 5B clearly dispel this notion. The possibility exists that the markedly unstable H2A.Z-H3.3-containing nucleosomes observed *in vivo* [133] are either heavily post-translationally modified and/or may simply represent a particle already in the process of unfolding, such as those described in [53]. This may result from their association with a yet unidentified chromatin remodelling complex.

Furthermore, the accumulation of H3.3 with age in vertebrate non-dividing tissues, described at the beginning of this section and which, in some instances, can represent up to 60% or more of the total H3 contents of chromatin, make a nucleosome-destabilizing role for this variant highly unlikely.

### 5.2. CenH3: True Histone Epigenetics with Controversial Nucleosome Organization

CenH3 (aka CENP-A) is one of the less evolutionarily conserved of all the members of the histone H3 family [34], yet—and very intriguingly—it represents the best example of a true epigenetic role for a histone variant [34,136]. Despite the high extent of its sequence variability, the ability of this protein to form neo-centromeres in human cells in a way that it is independent of an underlying α-satellite DNA sequence is quite remarkable [34]. Ubiquitination of CENP-A K124 has been recently shown to play a critical role in the deposition of CENP-A at the heterochromatic centromeric regions [137].

The structural organization of the CenH3-containing nucleosome has been quite controversial [138], a fact that may reflect the large extent of interspecific amino acid sequence variability of this histone in different organisms, as well as the specific PTMs associated to this variant. A very unusual nucleosome organization, consisting of half of the histone complement of a canonical nucleosome (hemisome), was initially reported by using a combination of crosslinking followed by immuno-precipitation with CenH3 specific antibodies in *Drosophila melanogaster* cells. [139]. In this organization, a heterotypic tetramer consisting of H2A.-H2B-H3 and H4 is associated with an approximately 120 bp of DNA. Furthermore, in contrast to nucleosomes consisting of canonical histones in which the DNA is wrapped in a left-handed superhelical conformation, in the CenH3 hemisomes the DNA was wrapped in a right-handed orientation [140,141]. The ability of the CenH3 variant to impart to the nucleosome this unusual organization may explain why the critical centromeric structure is more dependent on this variant rather than on the underlying centromere DNA sequences [34]. However, the hemisome structure has been much disputed, and alternative structures have been proposed. One such alternative model proposes that the intrinsic structure of the CenH3-H4 tetramer within an octameric nucleosome consisting of left-handed DNA is ultimately responsible for the uniqueness of this nucleosome [142]. An octameric structure was also reported from crystallographic analysis using human CenH3. However, in this instance, and similarly to what was observed for H2A.Bbd-containing nucleosomes only, 121 bp of DNA could be resolved in the crystallographic analysis–suggesting the flanking 13 bp DNA at the entry and exit of the nucleosome had a flexible organization [143]. More recent *in vitro* reconstitution experiments, using budding yeast CenH3 (Cse4) and the cognate 80 bp centromeric CDEII, provide support to the CenH3/H4/H2A/H2B tetramer hemisome conformation [144] which *in vivo* appears to wrap this DNA element in either orientation [145]. However, whatever the final conformation of CenH3 nucleosome, it appears to depend on its further interaction with other proteins of the centromeric complex, such as CENP-C [146].

## 6. Histone H1 Variants: Cell Differentiation and Developmental Histone Regulators of Chromatin Folding

**Figure 6:**
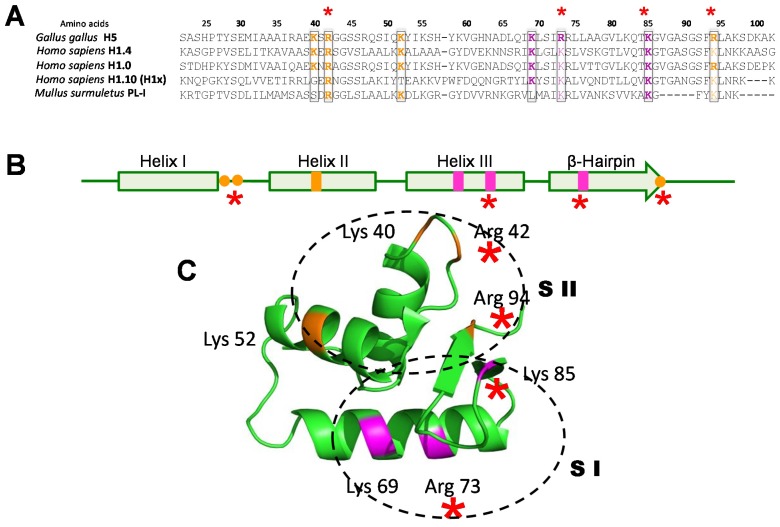
(**A**) Amino acid sequence alignment of the WHD regions of several linker histones. The amino acids corresponding to the first and second sites of interaction of this domain with DNA [154] in the chromatosome are highlighted in orange and magenta (respectively). The amino acid numbers refer to their position in the histone H5 sequence; (**B**) Schematic representation of the secondary structure of the WHD. The sites corresponding to the first and the second histone—DNA interacting domains are in the same colours as in (**A**); (**C**) The tertiary structure of the WHD of chicken erythrocyte histone H5 as determined by crystallographic analysis [155], showing the regions and amino acids corresponding to the first (SI) and second (SII) sites of interaction with DNA. The red asterisks highlight the minimal ionic interaction sites that appear to be indispensable for this domain to perform its function in vertebrate linker histones.

Globally, linker histones can provide neutralization of the excess linker DNA charge, thus enhancing the folding of the string of nucleosomes in the chromatin fiber, and act in this way in a transcriptionally repressive manner (although they have been described as also having an activating function in particular instances [147]). This charge shielding function is shared with the highly charged N-terminal tails of the core histones, which in the case of H1 histones is mainly contributed by their charged C-terminal domain [148]. In metazoans, the acquisition of a WHD [149] confers on these proteins the ability to specifically recognize and bind to DNA cruciform-like structures [150,151]. Such DNA organization is analogous to that adopted by the linker DNA at the entry and exit of the nucleosome, through the interaction of two highly conserved binding sites in the WHD (Figure 6). Unlike the HFD of core histones, the WHD of linker histones does not constitute a genuine protein dimerizing domain; however, it has the potential to dimerize [152]—a fact that may enhance its contributing role in the folding of chromatin fiber [153].

To emphasize the structural conservation of the WHD in the histone H1 variants (Figure 6), we show several structural features of this domain by comparing the replication-dependent human somatic histone H1 types (histone H1.1–H1.5) represented by H1.4 to the two replication-independent human types: H1.0 (previously H1°) and H1.10 (previously H1x), as well as to two extreme examples of H1 histones that accumulate in terminally differentiated cells of vertebrates: histone H5 of chicken erythrocytes, and PL-I of the sperm of the *Mullus surmuletus* [156]. Histone H1.4 is one of the most abundant types in the cell. The functional roles of H1.0 and H1.10 remain elusive. However, H1.0 is known to accumulate in differentiated non-dividing cells with aging and H1.10 was initially described to be present in chromatin regions that are resilient to nuclease digestion [157]. Histone H5 is a highly specialized H1 variant, which is present in the nucleated erythrocytes of vertebrates [158]. PL-Is are arginine and lysine rich (protamine-like) proteins found in the sperm of some invertebrate [159,160] and vertebrate organisms [156] where, like protamines, they displace most of the somatic histones [45]. Despite the amino acid sequence variability observed amongst the different WHDs (Figure 6A), the secondary (Figure 6B) and tertiary (Figure 6C) structures predicted using bioinformatics indicate a highly conserved organization; they are almost indistinguishable from the crystallographic structure initially determined for the WHD of chicken erythrocyte histone H5 [155]. Interestingly, this structural preservation and the evolutionarily conserved basic amino acids therein maintain the integrity of the two SI and SII sites [154] that define the intrinsic binary DNA binding characteristics of this domain. Moreover, the comparison of WHDs from such diverse linker histone-related proteins allows for the reduction the number of essential binding amino acids to four (see asterisks in Figure 6), whereas in the case of *M. surmuletus* PL-I, the highly conserved [G(V/T)GASGS] β-hairpin appears to be dispensable when nucleosomes are absent. Thus, the characteristic presence of a WHD in the different histone H1 variants appears to be involved in maintaining the divalent interaction that allows them to interact with neighbouring DNA molecules [150,156] under different settings.

Except for the main lysine-rich somatic types (H1.1-H1.5) whose N- and C-terminal domains exhibit sequence micro-heterogeneity [161], the rest of the members of this protein family exhibit an enormous sequence and size variability in these regions [148] in comparison to the much conserved WHD (Figure 6). The role of the somatic histone H1 microheterogeneity of the somatic H1.1-H1.5 types is not very well understood. Despite their low level of sequence variability [54], their functions do not seem to be completely redundant and exhibit some extent of preferential genome distribution [162,163], different chromatin affinity [164], and binding dynamics [165].

The replication-independent linker histones H1.0 and H1.10, often preferentially present at different stages of cell differentiation, and H5 and PL-I, erythrocyte, and sperm-specific histones, have highly compositionally variable N- and C- terminal domains. Histone H5 variants exhibit an increased arginine content, presumably to enhance their heterochromatinization ability. Sperm-specific PL-Is exhibit a larger size diversity in their N- and C-terminal regions, as well as an increase in the amounts of basic residues (arginine/lysine) present in these regions. Therefore, while somatic histone H1 microheterogeneity may account for the modulation of chromatin structure required to accommodate the metabolic changes that take place at different developmental stages, more extreme variations of the N- and C- terminal domains appear to be critical to maintaining the terminally differentiated stages.

## 7. The Structure and the Function. A Brief Look at the Transcription Start Site

**Figure 7:**
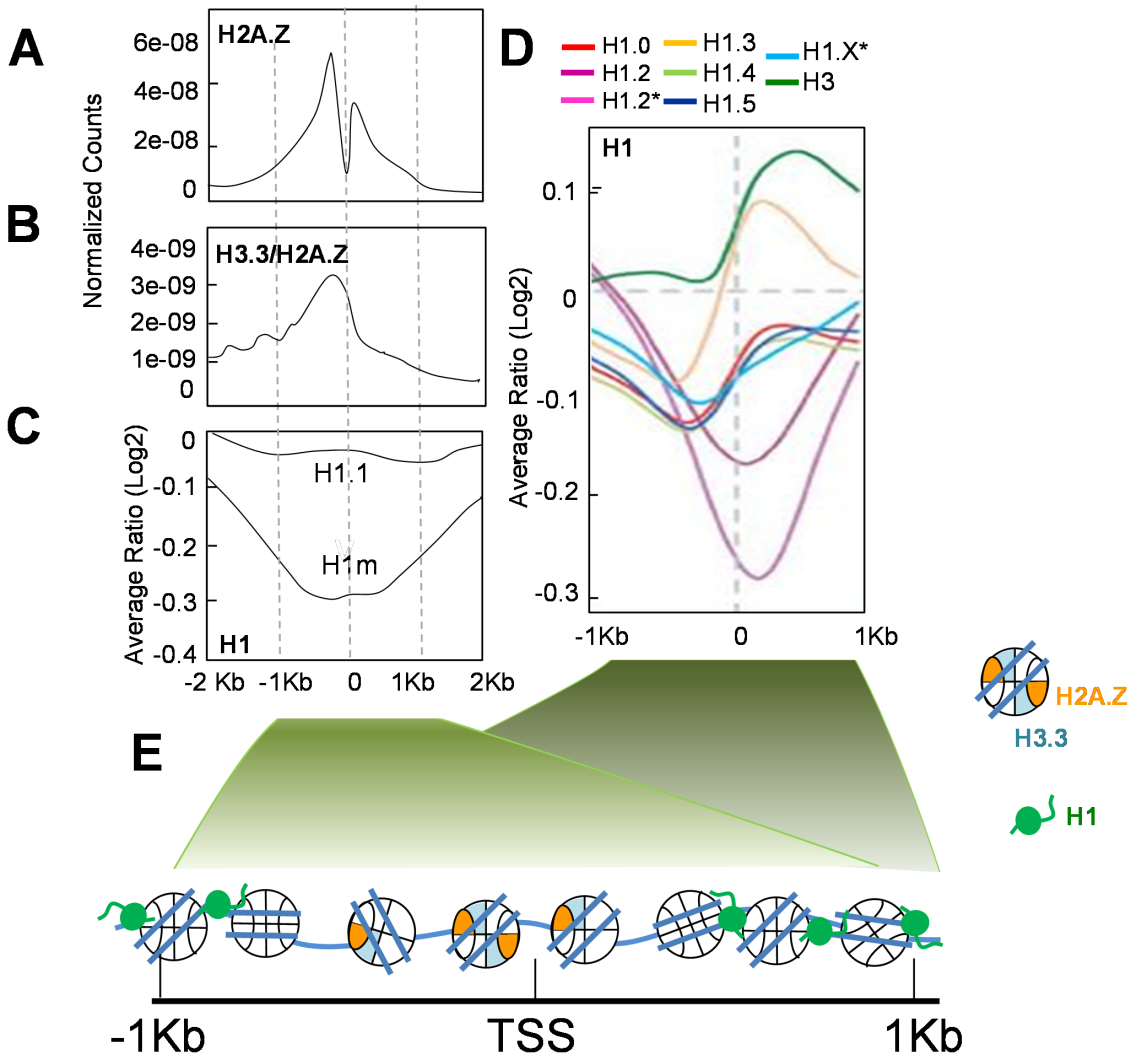
Histone occupancy profiles around TSS. (**A**) Averaged H3.3/H2A.Z nucleosome positioning [70]; (**B**) Profile of histone H2A.Z distribution in high and medium transcriptionally active genes [167]; (**C**) Histone H1 subtypes distribution at TSS for transcribed and non-transcribed genes. H1.1 corresponds to the distribution observed for this subtype as compared to H1m, corresponding to the average for H1.2-H1.3-H1.4-H1.5 [162]; (**D**) Depletion of histone H1-tagged subtypes at promoters. The asterisks indicate analysis conducted using the endogenous histones [163]; (**E**). Schematic representation of histone variants H3.3 and H2A.Z, and histone H1 distribution around gene promoters.

One of the most extensively characterized genome-wide distributions of histone variants of chromatin is in gene promoter regions, particularly around the TSS [166] (Figure 7).

It has been shown that H2A.Z is an important marker for the nucleosomes immediately preceding and following the TSS [167] (Figure 7A)—particularly in those genes that are poised for transcriptional activation. Furthermore, a co-habitation of this variant within H3.3 exists around these regions [70] (Figure 7B), which are further characterized by a histone H1 depletion [162] (Figure 7C). As described in earlier sections, the absence of histone H1, which would ensure an open conformation around these regions, is most likely the result of the impairment of its binding to H2A.Z-containing nucleosomes. The structural implications, if any, of the dual presence of H2A.Z and H3.3 in the nucleosomes preceding the TSS (Figure 7B) remains to be established (as mentioned above, in the histone H3.3 section). Both H2A.Z and H3.3 variants, however, have been shown to play a very important role in transcription initiation where—in conjunction with the chromatin remodelers RSC, SWR, and HIRA—they are targeted to the nucleosomes immediately adjacent to the TSS, in a histone acetylation-dependent way [124]. This results in a dynamic chromatin environment which allows for multiple rounds of transcription, and is amenable to transcription elongation [124] The relevance of the distribution of the different H1 types around the promoter regions is equally important (Figure 7D). While most of the somatic linker histone types (H1.1, H1.3, H1.4, and H1.5) are clearly depleted from the regions immediately preceding the TSS, only H1.2 is clearly depleted from the region immediately following the TSS (Figure 7D) [163]—implying a distinctive functional role.

Figure 7E provides a cartoon representation of the histone variant landscape and H1 type distribution around promoter regions. This unique histone variant distribution provides a good example of how some of them, particularly H2A.Z, can operate as an epigenetic landmark for the activation of transcriptionally poised genes. Although the detailed mechanism for the cellular transmission of such epigenetic information is still not well understood, it highlights some of the remaining important challenging questions on the true epigenetic role of histone variants. The enhanced affinity of the H2A.Z-H2B dimer by the H3-H4 tetramer [60] might contribute to ensure the fidelity of the transmission of the H2A.Z-containing nucleosome at a particular site during DNA replication. Yet, as with many of the other histone variants, experimental *in situ* evidence for such predictions is still missing.

## 8. Conclusions

Of all histones variants, histone H2A family represent the most abundant class [75]. Their structural and functional characteristics are mainly exerted through their N-and C-terminal tails [31], with the length of the C-terminal end playing a critical role in nucleosome stability—as evidenced by the low stability of H2A variants such as H2A.Z.2.2 and H2A.Bbd that are truncated at this region. Variations in their C-terminal domains also play important structural roles that globally result in an impairment of histone H1 binding; however, how these roles are epigenetically transmitted and inherited is less clear. Yet, as pointed out above, the enhanced affinity of the H2A.Z-H2B dimer for the histone core may generate a nucleosome imprint that maintains not only its composition but ensures the absence of H1. Additionally, some of the variants like H2A.Z and macroH2A have been shown to act both as activators and as repressors of transcription. The structural details surrounding this functional duality are not yet clearly understood, although it is likely that histone PTMs play a role in it.

In the case of histone H3 variants, despite the controversial role of CenH3 in the nucleosome organization, the mechanism of its epigenetic inheritance is quite well understood. This mechanism is not as clear in H3.3, but a post-translationally-mediated process that involves the specific methylation of H3.3 at lysine 4 has been invoked [168]. It would be fascinating to pursue an understanding of the mechanisms involved in the accumulation of this variant, as well as of H1.0, with aging. It is actually amazing that it has taken over 30 years to rediscover this very interesting problem [9]!

In closing, it has long been known that two of the most distinctive global structural signatures of transcriptionally active chromatin are histone acetylation and depletion of histone H1 [169]. Some of the previous sections have highlighted a prevalent structural feature (*i.e*., preventing histone H1 binding) directly contributed by several histone variants during their replacement of canonical histones and thus providing a mechanism for H1 depletion. Furthermore, global histone acetylation does not, *per se*, result in a histone H1 deficiency [170] but alters its capacity of binding back upon its eviction from the nucleosome [171]. In this way, it is tempting to speculate that histone variants and histone acetylation could act synergistically to create the facilitator “open” chromatin structures responsible for what, over the years, has been taken as one of the most important hallmarks of transcriptionally active genes: nuclease hypersensitivity [169].

## Abbreviations

CDEII: Centromere DNA element II
ChIP: Chromatin immune-precipitation
DNA-PK: DNA-dependent protein kinase
CTD: C-terminal domain
DAXX: Death-domain associated
EST: Expressed sequence tag
FRAP: Fluorescence-recovery after photobleaching
GFP: Green fluorescent protein
HFD: histone fold domain
HIRA: Histone cell cycle regulation defective homolog A
NCP: nucleosome core particle
NHD: Non-histone domain
NRL: nucleosome repeat length
SDS-PAGE: sodium dodecyl sulfate-polyacrylamide gel electrophoresis
NTD: N-terminal domain
PTMs: Post-translational modifications
RSC: Remodel structure of chromatin
SWR: Sick with RSC/Rat1 complex
TSS: Transcription start site
WHD: winged helix domain
